# Progressive Paraplegia Due to Spinal Dural Arteriovenous Fistula: A Rare but Treatable Diagnosis That Should Not Be Missed

**DOI:** 10.7759/cureus.5893

**Published:** 2019-10-11

**Authors:** Sajid Hameed, Bushra Taimuri, Marvi Sajid, Farah Siraj, Ayeesha Kamal

**Affiliations:** 1 Neurology, Aga Khan University, Karachi, PAK; 2 Anatomy, United Medical and Dental College, Karachi, PAK; 3 Internal Medicine, Chandka Medical College Hospital, Larkana, PAK

**Keywords:** sdavf, spinal dural arteriovenous fistula, paraplegia, spinal vascular malformations, spinal arteriovenous malformations

## Abstract

Spinal dural arteriovenous fistula (SDAVF) is the most common spinal vascular anomaly. It should always be considered in the differential diagnoses in a patient with progressive paraplegia or quadriplegia. We present a case of an elderly gentleman presenting with progressive paraplegia. The diagnosis was delayed as the previous physicians and radiologists missed the underlying key features of SDAVF on magnetic resonance imaging of the spine. Every neurologist and radiologist should be aware of these signs as SDAVF is mostly a treatable condition.

## Introduction

Spinal dural arteriovenous fistula (SDAVF) is the most common spinal vascular anomaly. It should always be considered in the differential diagnoses in a patient with progressive paraplegia or quadriplegia. We present a case of an elderly gentleman presenting with progressive paraplegia. The diagnosis was delayed as the previous physicians and radiologists missed the underlying key features of SDAVF on magnetic resonance imaging (MRI) of the spine. Every neurologist and radiologist should be aware of these MRI features as SDAVF is mostly a treatable condition.

## Case presentation

A 70-year-old gentleman presented with a 12-month history of progressive bilateral lower limb weakness and numbness. He also developed constipation and urinary retention over the two weeks preceding presentation. There was no history of fever, backache, trauma, weight loss, or cough. Medical history was significant for type-2 diabetes mellitus and hypertension. For these symptoms, he consulted multiple physicians at different hospitals and underwent three magnetic resonance imaging (MRI) of the spine, which the radiologists reported as cervical spondylosis, transverse myelitis, and cervical syrinx, respectively. He was managed with high-dose intravenous steroids (methylprednisolone 1000 mg daily for five days) with tapering oral steroids, multivitamins, and coenzyme Q but his weakness gradually worsened.

On neurological examination, higher mental functions were intact. Cranial nerves examination was within normal limits. Pupils were bilateral 3 mm in diameter with normal pupillary reflex to light and accommodation. The fundus examination was unremarkable. Muscle bulk and muscle tone were normal in all four limbs. Muscle strength was normal in upper limbs while it was reduced in the lower limbs bilaterally, with the Medical Research Council (MRC) grade of 3/5 in both proximal and distal muscle groups. Deep tendon reflexes were normal in all four limbs except the ankle reflexes, which were reduced (1+) bilaterally. Babinski’s reflex was present bilaterally. Pinprick sensations were reduced below the level of T7. Proprioception testing was abnormal in lower limbs bilaterally.

**Figure 1:**
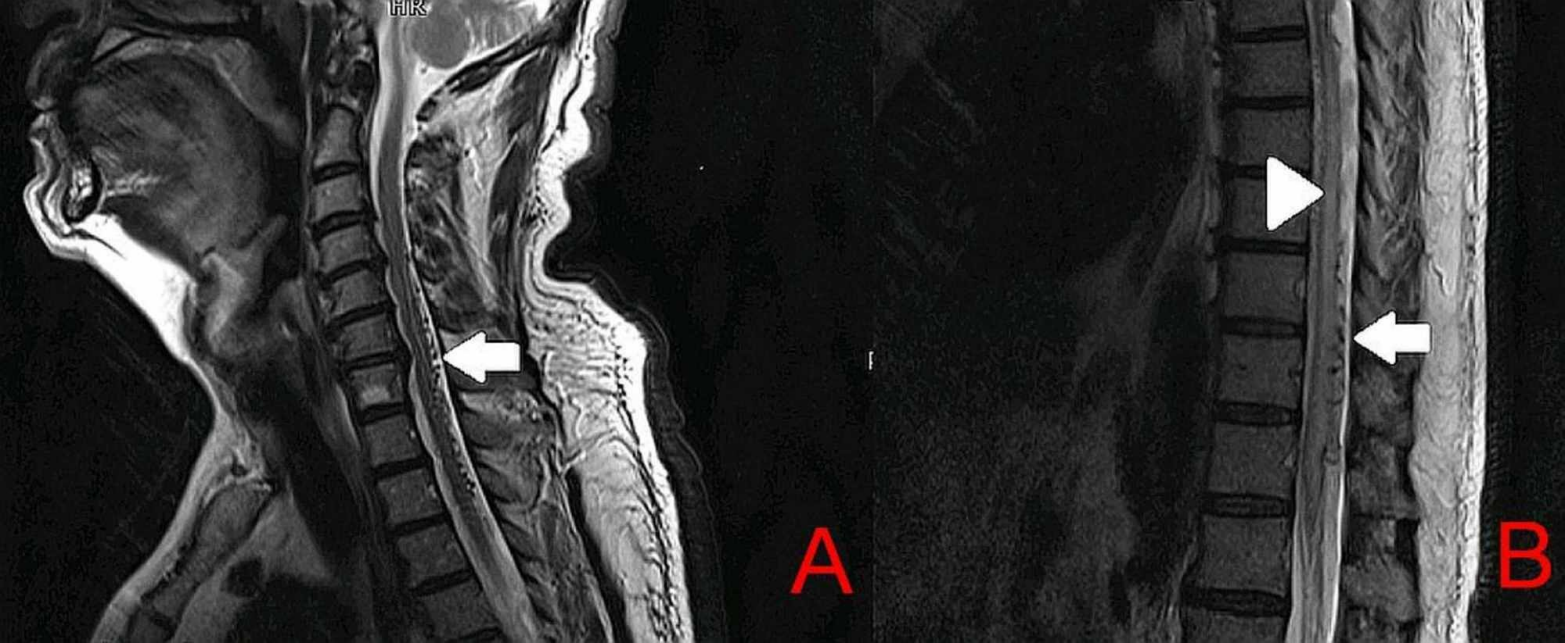
T2 MRI of the cervical spine (A) and thoracic spine (B) White arrows indicate vessel flow voids dorsally. Arrowhead indicates T2 hyperintense signals and cord edema MRI - magnetic resonance imaging

Another MRI of the spine was performed (Figure 1). The T2-weighted MRI images revealed patchy edema in the spinal cord extending from the C4 vertebral body up to the conus along with vessel flow voids dorsally. These findings were suggestive of spinal dural arteriovenous fistula (SDAVF). Although these findings were also present in the previous MRI scans, they were missed on reporting.

**Figure 2:**
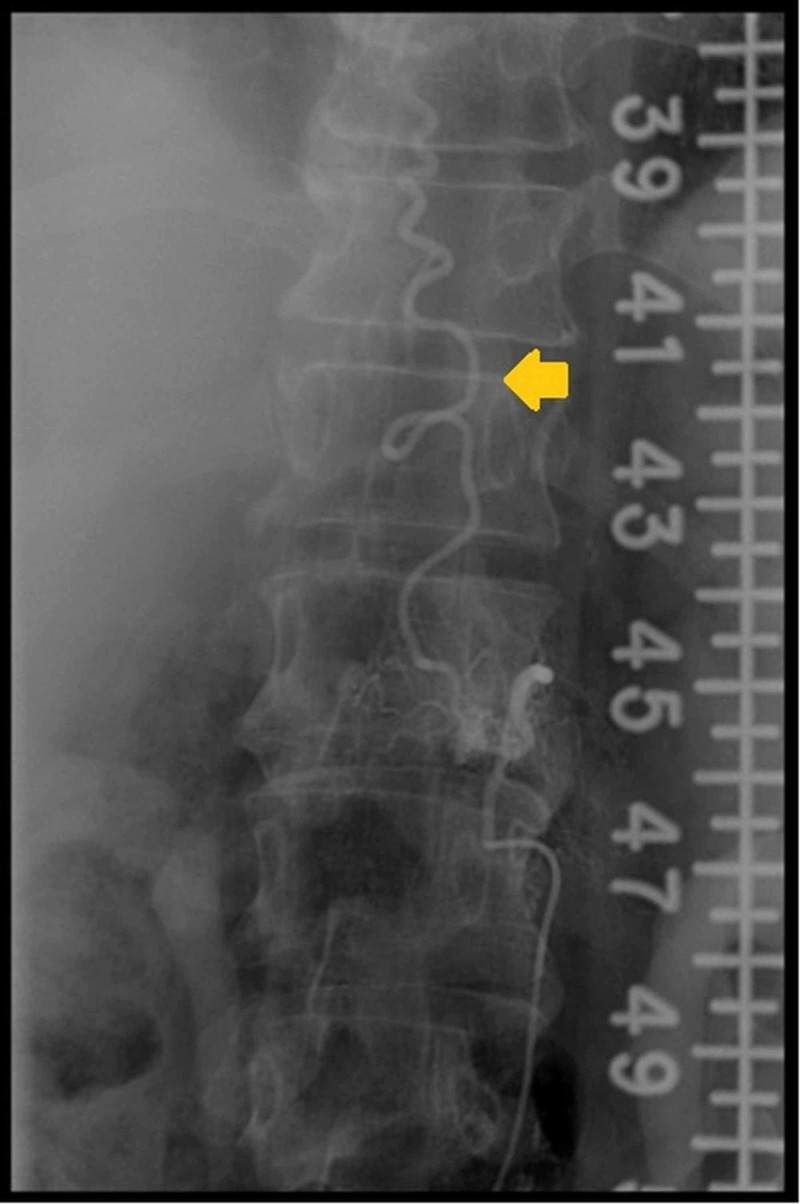
Typical angiographic features of spinal dural arteriovenous fistulas (SDAVFs) The yellow arrow shows the perimedullary vein

The diagnosis of SDAVF was subsequently confirmed on digital subtraction angiography (Figure 2) and the fistula was embolized using histoacryl glue and lipoidal mixture. He was stable post-procedure and his weakness improved (MRC grade 4/5) within the next 48 hours.

## Discussion

The spinal dural arteriovenous fistula was first described in 1926 by Foix and Alajouanine [1]. It is the most common vascular malformation of the spinal cord, albeit still rare, with an annual incidence of only five to ten cases per million [2]. SDAVFs are common in middle-aged and elderly populations with a reported mean age of approximately 60 years. Males are about six times more commonly affected than females. The exact etiology of SDAVF is largely unknown but it is mostly an acquired condition. More than 75% of the SDAVFs are located from the mid-thoracic to the upper lumbar spine. The presence of a fistula leads to arterialization of the spinal veins resulting in venous hypertension, venous engorgement, and if untreated will lead to cord ischemia and infarction [1].

Diagnosis of SDAVF is often missed leading to diagnostic delay, as in our case. To our surprise, the literature reveals an average delay of around 15-24 months from the symptom-onset to the confirmation of the diagnosis of SDAVF [3-4]. This delay in diagnosis results in an increase in the severity of motor and sensory symptoms, with 10-30% of the cases are wheel-chair bound at the time of diagnosis [1]. Apart from the motor weakness, worsening sphincter dysfunction and sexual problems adversely affect the quality of life.

Spinal MRI is often the first choice of investigation. It not only excludes the common causes of paraplegia but also reveals characteristic appearances suggestive of SDAVF. These include (1) T2 hyperintense signals within the cord, (2) spinal cord expansion, and (3) vessel flow voids on the dorsal and/or ventral aspect of the cord. In chronic cases of SDAVF, there may be some T1 postcontrast enhancement or spinal cord atrophy [1]. When these MRI features are missed on initial MRI spine and radiologists do not suspect a vascular etiology, a mean diagnostic delay of 281 days from the initial MRI scan was seen in the diagnosis of SDAVF as compared to only 22 days when a vascular etiology was suspected in the initial MRI reporting [5]. Digital subtraction angiography confirms the diagnosis when a clinical and/or radiological suspicion is made for SDAVF.

Multiple classifications of SDAVF have been proposed over the years based on the angiographic features and have been the source of debate among neuroscientists. In 1971, Di Chiro G classified spinal arteriovenous shunts into three types based on their angiographic appearances: type I (single coiled vessel), type II (glomus), and type III (juvenile). The type I corresponded to spinal dural arteriovenous fistulas (AVFs), while types II and III corresponded to the intradural arteriovenous malformations (AVMs) [6]. In 1987, Rosenblum et al. added a type IV spinal arteriovenous shunt (intradural direct arteriovenous fistula) into the classification [7]. In 1993, Mourier et al. renamed the type IV from intradural AVFs to perimedullary AVFs. They further subdivided the type IV into three subtypes depending upon the number of feeding arteries and the size of AVF [8]. Further newer classifications are proposed by Rodesh in 2002, by Geibprasert in 2008, and by Rangel-Castilla in 2011 [9]. Discussing each classification will be beyond the scope of this article. The first three classifications are summarized in Table 1.

**Table 1:** Different classifications of spinal dural arteriovenous fistulas AVF - arteriovenous fistula; AVM - arteriovenous malformation

Classification	Type	Academic name	
Di Chiro (1971) [5]	I	Single coiled vessel type	
	II	Glomus type	
	III	Juvenile type	
Rosenblum (1987) [6]	I	Dural AVF	
	II	Intramedullary glomus AVM	
	III	Intramedullary juvenile AVM	
	IV	Intradural direct AVF	
Mourier (1993) [7]	I	Dural AVF	
	II	Intramedullary glomus AVM	
	III	Intramedullary juvenile AVM	
	IV	Perimedullary AVF	
	Type IV subtypes:	I	A single feeder and small AVF
		II	Multiple feeders and medium AVFs
		III	Multiple feeders and a giant AVF

SDAVF has a variable prognosis but almost 90% of the patients experience either stabilization or improvement in their symptoms following treatment [2]. The treatment involves either microsurgery or endovascular embolization to close the abnormal vascular connection. Since SDAVF is largely a reversible condition if diagnosed early, the neurologists and radiologists should be aware of the MRI findings to avoid unnecessary delays in the diagnosis and treatment of SDAVF [10].

## Conclusions

SDAVF should be considered in the differential diagnoses in a patient with progressive paraplegia or quadriplegia. MRI of the spine typically shows T2-hyperintense signals in the spinal cord, cord expansion, and vessel flow voids. SDAVF is a treatable condition with good prognosis if diagnosed early.
